# Fc engineered ACE2-Fc is a potent multifunctional agent targeting SARS-CoV2

**DOI:** 10.3389/fimmu.2022.889372

**Published:** 2022-07-28

**Authors:** Bruce D. Wines, Liriye Kurtovic, Halina M. Trist, Sandra Esparon, Ester Lopez, Klasina Chappin, Li-Jin Chan, Francesca L. Mordant, Wen Shi Lee, Nicholas A. Gherardin, Sheila K. Patel, Gemma E. Hartley, Phillip Pymm, James P. Cooney, James G. Beeson, Dale I. Godfrey, Louise M. Burrell, Menno C. van Zelm, Adam K. Wheatley, Amy W. Chung, Wai-Hong Tham, Kanta Subbarao, Stephen J. Kent, P. Mark Hogarth

**Affiliations:** ^1^Immune therapies Laboratory, Burnet Institute, Melbourne, VIC, Australia; ^2^Life Sciences, Burnet Institute, Melbourne, VIC, Australia; ^3^Department of Immunology and Pathology, Central Clinical School, Monash University, Melbourne, VIC, Australia; ^4^Department of Clinical Pathology, The University of Melbourne, Parkville, VIC, Australia; ^5^Department of Microbiology and Immunology, The Peter Doherty Institute for Infection and Immunity, The University of Melbourne, Melbourne, VIC, Australia; ^6^Infectious Diseases and Immune Defence Division, The Walter and Eliza Hall Institute of Medical Research, Parkville, VIC, Australia; ^7^Department of Medical Biology, The University of Melbourne, Melbourne, VIC, Australia; ^8^Department of Medicine, Austin Health, The University of Melbourne, Melbourne, VIC, Australia; ^9^Department of Medicine, Royal Melbourne Hospital, The University of Melbourne, Parkville, VIC, Australia; ^10^Department of Microbiology, Monash University, Clayton VIC, Australia; ^11^Department of Allergy, Immunology and Respiratory Medicine, Central Clinical School, Alfred Hospital, Melbourne, VIC, Australia; ^12^Australian Research Council Centre for Excellence in Convergent Bio-Nano Science and Technology, The University of Melbourne, Melbourne, VIC, Australia; ^13^World Health Organization (WHO) Collaborating Centre for Reference and Research on Influenza, The Peter Doherty Institute for Infection and Immunity, The University of Melbourne, Melbourne, VIC, Australia; ^14^Melbourne Sexual Health Centre and Department of Infectious Diseases, Alfred Hospital and Central Clinical School, Monash University, Melbourne, VIC, Australia

**Keywords:** coronavirus, SARS-CoV-2, COVID-19, ACE2-Fc, neutralization, antibody effector function, ADCC, complement

## Abstract

Joining a function-enhanced Fc-portion of human IgG to the SARS-CoV-2 entry receptor ACE2 produces an antiviral decoy with strain transcending virus neutralizing activity. SARS-CoV-2 neutralization and Fc-effector functions of ACE2-Fc decoy proteins, formatted with or without the ACE2 collectrin domain, were optimized by Fc-modification. The different Fc-modifications resulted in distinct effects on neutralization and effector functions. H429Y, a point mutation outside the binding sites for FcγRs or complement caused non-covalent oligomerization of the ACE2-Fc decoy proteins, abrogated FcγR interaction and enhanced SARS-CoV-2 neutralization. Another Fc mutation, H429F did not improve virus neutralization but resulted in increased C5b-C9 fixation and transformed ACE2-Fc to a potent mediator of complement-dependent cytotoxicity (CDC) against SARS-CoV-2 spike (S) expressing cells. Furthermore, modification of the Fc-glycan enhanced cell activation *via* FcγRIIIa. These different immune profiles demonstrate the capacity of Fc-based agents to be engineered to optimize different mechanisms of protection for SARS-CoV-2 and potentially other viral pathogens.

## Introduction

Recent history has seen regular deadly zoonotic coronavirus spillover events with the emergence of severe acute respiratory syndrome coronavirus (SARS-CoV) in 2002 (1), Middle East respiratory syndrome (MERS) coronavirus in 2012 (2) and SARS-CoV-2 in December 2019 (3). SARS related coronaviruses are found in bats throughout Southeast Asia (4) and the serology of people living in proximity to a Rhinolophus spp bat colony suggests these zoonotic infections are not uncommon (5). Since the publication of the SARS-CoV-2 genome in January 2020 (3) there has been rapid development and deployment of vaccines for SARS-CoV-2 (6) and the clinical development of multiple SARS-CoV-2 spike specific neutralizing monoclonal antibodies (mAbs) from convalescent patients or animals, reviewed in (7).

Evolution of the SARS-COV-2 spike protein has selected for increased transmissibility, for example by increased affinity for host cells (8), with the emergence and then dominance of many new variants of concern (VOC) including Alpha, B.1.1.7; Beta, B.1.351; Gamma, P.1; Delta, B.1.617.2 (9) and most recently, Omicron, B.1.1.529 (WHO) that impact the neutralization efficacy of antibodies generated against the spike antigen of earlier strains (10). This includes profound escape from neutralization by some mAbs (11–15) and significant loss of neutralization activity of convalescent sera (13, 16, 17) and of humoral responses to first generation vaccines (12, 18, 19) reviewed in (10). Reinfection by neutralization-escape variants (20, 21) and break-through infection in vaccinees is now a feature of the pandemic (13, 19). Furthermore, protective antibody responses in humans are largely restricted to specific coronavirus species since few Abs to SARS-CoV-2 receptor binding domain (RBD) cross-neutralize SARS-CoV or MERS-CoV (22) but the recent identification of spike-specific broadly neutralizing mAbs may be a key to future pan-beta-coronavirus pandemic preparedness (23). Overall, despite increased surveillance and biosecurity (24) and the development of SARS-CoV-2 vaccines and mAbs, a critical vulnerability to variants of concern (VOC) and future pandemic novel coronaviruses persists. There is a need for prophylactic and therapeutic approaches that are more broadly effective against Sarbecoviruses.

Decoy proteins, based on the host entry receptor, inhibit viral entry, and achieve cross neutralization of multiple virus species or strains (25–31). Angiotensin-converting enzyme 2 (ACE2), is the principal entry receptor for the major human pathogenic coronaviruses, SARS-CoV and SARS-CoV-2 (32, 33), as well as the human endemic coronavirus NL63 (34). ACE2 is a transmembrane carboxypeptidase (35) with the ectodomain comprised of a catalytic domain, as well as a collectrin domain likely involved in dimerization (36). It normally plays a role in cardiovascular homeostasis by cleaving angiotensin II, the key agonist of the renin–angiotensin–aldosterone system (RAAS) that regulates blood pressure and electrolytes (37, 38). SARS-CoV-2 entry into host cells is blocked by the recombinant soluble ACE2 catalytic ectodomain in its native form (39, 40) or when engineered for higher avidity (31, 41, 42) or affinity (28, 29, 31, 41–45) for the ancestral spike, which has thus far been retained against later VOC (28, 29, 31, 41). Enzyme inactive forms of antiviral ACE2 decoy proteins have also been developed (26, 46, 47). However, ACE2 enzymatic activity, by cleaving angiotensin II, is protective in lung injury models and may therefore be beneficial to retain in an ACE2-based biological for COVID19 (38, 39, 48, 49).

To improve virus neutralization potency or pharmacokinetic properties of SARS-CoV-2 decoys, the ACE2 ectodomain, with or without the collectrin domain, has been fused to the Fc portion of IgG (26, 28, 29, 42–47, 50–55), which results in increased neutralisation potency by bivalency and increased serum half-life (56). In mAb studies using *in vivo* SARS-CoV-2 challenge models, Fc-dependent immune effector functions, which include antibody-dependent cellular cytotoxicity (ADCC), phagocytosis and clearance of viruses, bolstered protection against infection and pathology above that provided by neutralization alone (57–61). Similarly, ACE-Fc decoys have demonstrated protective activity in human ACE2 transgenic mouse models (29, 49, 62) and in hamsters (28) challenged with SARS-CoV-2.

We report the development of multifunctional antiviral proteins by applying novel mutations of the Fc and glycan modification to manipulate the Fc component of ACE2-Fc which resulted variously in increased virus neutralization, complement directed killing and activation of FcγRIIIa.

## Materials and methods

### Constructs and proteins

*-trACE2* and *trACE2-Fc* Truncated ACE2 (trACE2) comprised the catalytic portion of the ACE2 ectodomain and a sequence encoding trACE2 (aa 19-615, Accession BAB40370) in pHLsec (63) was a gift from Merlin Thomas (64). This trACE2 sequence was fused to a synthetic DNA for human IgG1 Fc (Accession AXN93652.1) in pcDNA3.4 (ThermoFisher) with an encoded linker sequence D^615^-GSGSGSG-T^223^, where D^615^ is the last residue of ACE2 and T^223^ (Eu numbering) is the fusion point to IgG1-Fc on the amino terminal side of the Fc core hinge containing the inter-heavy chain disulfides (for full amino acid sequences see **Supplementary Text**). -*flACE2-Fc* comprised the full-length ACE2 ectodomain fused to human IgG1-Fc. The incorporation of a synthetic DNA encoding the collectrin domain (GeneArt, ThermoFisher) formed a full length ACE2 ectodomain encoding sequence (aa 19-740) fused to the human IgG1-Fc *via* a linker with the sequence S^740^-GGGGS-T^223^, where S^740^ is the last residue of ACE2 and T^223^ is the fusion point to IgG1-Fc. *EflACE2-Fc.* EflACE2-Fc was equivalent to the flACE2-Fc, except it incorporated the three mutations, T27Y, L79T, N330Y reported as sACE2.v2.4 and having enhanced affinity for SARS-CoV-2 spike RBD (43). The EflACE2-Fc construct was a synthetic DNA (GeneArt) in pcDNA3.4 (ThermoFisher). The mutations H429F, H429Y and E430G in the Fc were introduced using cleavage at a unique Afe *I* (New England Biolabs) site within codons for E430-L432 and the insertion of appropriate mutagenic oligos with NEBuilder according to the manufacturer’s instructions (NEB).

ACE2-Fc protein expression used transient transfection of Expi293 cells (Thermo Fisher Scientific). The supernatant of Expi293 transiently transfected for the expression of ACE2-Fc was extensively dialysed against 10mM TrisHCl pH 8 and applied to a High-Q column (BioRad Laboratories). Bound proteins were eluted with the indicated gradient to buffer A with 0.4 M NaCl and washed with 1 M NaCl. Fractions were examined by SDS-PAGE, fractions containing ACE2-Fc were pooled and concentrated using a 30 kDa cut-off filtration device (Merck) and separated by SEC using a Superose 6 column (GE Lifesciences). Lamelli native PAGE (150V, 2.5 h, 4°C), was performed according to (65).

Recombinant Spike receptor binding domain (RBD; aa328-514, GenBank: MN908947.3) of SARS-CoV-2 Wuhan strain was produced with the N-terminal Fel d 1 leader sequence and C-terminal biotin ligase (BirA) AviTag and a hexahistidine affinity tag (Hartley et al., 2020). Specific mutations were introduced in this construct to generate SARS-CoV-2 variant RBD proteins, representing those from three lineages of concern: B.1.351 (beta; N501Y, E484K, K417N), P.1 (gamma; N501Y, E4848K, K417T) and B.1.167.2 (delta; T478K, L452R). The DNA constructs were codon-optimized for *H. sapiens* and cloned into a pCR3 expression vector. Plasmid DNA was purified from *E. coli* by Maxiprep (Zymo Research, Irvine, CA), and 30 μg DNA was transfected into Expi 293F cells using the Expi293 Expression system (Thermo Fisher, Waltham, MA). Supernatants from 25 ml cell cultures were collected 5 days post-transfection and purified by application to a Talon NTA-cobalt affinity column (Takara Bio, Kusatsu, Shiga, Japan) with elution in 200 mM Imidazole. Eluted proteins were then dialyzed against 10 mM Tris for 48 hours at 4°C.

#### Virus neutralization assays

Antiviral activity was determined using SARS-CoV-2 (CoV/Australia/VIC01/2020) in a microneutralization assay where cytopathic effect was titred to limiting dilution on Vero cells as described previously (64, 66).

#### Bio-layer interferometry

Measurements of the affinity of ACE2 proteins for S protein RBD (64) were performed on the Octet RED96e (FortéBio). All assays were performed at 25°C using anti-human IgG Fc capture (AHC) biosensor tips (FortéBio) in kinetics buffer (PBS pH 7.4 supplemented with 0.1% (w/v) BSA and 0.05% (v/v) Tween-20). After a 60 second (60s) biosensor baseline step, ACE2-Fc recombinant proteins (20 μg/mL) were loaded onto the AHC sensors by submerging sensor tips for 200s and then washing in kinetics buffer for 60s. For most ACE2-Fc recombinant proteins, association measurements were performed by dipping into a two-fold dilution series of SARS-CoV-2 spike RBD (64) from 16–250 or 500nM for 180s and dissociation was measured in kinetics buffer for 180s. For EflACE2-Fc WT a two-fold dilution series of 2 – 31 or 63nM was used. Sensor tips were regenerated five times using a cycle of 5s in 10 mM glycine pH 1.5 and 5 s in kinetics buffer. Baseline drift was corrected by subtracting the average shift of an ACE2-Fc-loaded sensor not incubated with SARS-CoV-2 spike RBD, and an unloaded sensor incubated with SARS-CoV-2 spike RBD. Curve fitting analysis was performed with Octet Data Analysis 10.0 software using a global fit 1:1 model to determine K_D_ values and kinetic parameters. Curves that could not be fitted were excluded from the analyses.

##### ACE2-Fc binding ELISA

ELISA plates were coated with 5μg/ml CoV-2 receptor binding domain fused to mIgGFc (RBD-Ig, RBD aa residues 334-527) and blocked with phosphate buffered saline (PBS) containing 0.05% (w/v) Tween-20 and 2% (w/v) bovine serum albumin (BSA). RBD-Ig was reacted with ACE2-Fc proteins diluted in ½ log titrations (1 hour, 25°C) followed by washing 5 times with PBS, 0.05% Tween-20. Bound ACE2-Fc was detected with sequential incubation with mouse anti-human IgG1-biotin (Thermo MH1515, clone HP6070, at 1μg/ml for 1 hour, 25°C), high sensitivity streptavidin-HRP (1/10,000 dil, 1 hour, 25°C, Pierce, Thermo Scientific) and TMB substrate.

#### ACE2-Fc and dimeric recombinant soluble (rs)FcγR binding by flow cytometry

The ACE2-Fc proteins or the anti-CD20 mAb, Rituximab, at 5µg/ml, or the indicated concentrations were incubated with Ramos cells expressing transfected spike proteins (Ramos-S cells) (67) at 5x10^6^ cells/ml in 25µl in fluorescence activated cell sorting (FACS) buffer- PBS containing 0.5% (w/v) BSA, 1mM glucose (PBS/BSA/G), for 30 min on ice. Cells were washed twice with FACS-buffer, incubated with APC conjugated anti-human IgG-Fc for thirty minutes on ice, washed again and resuspended in 25 µl of FACS-buffer.

Evaluation of the binding of dimeric rsFcγR was performed as described in (68). ACE2-Fc opsonized Ramos-S cells were resuspended in 0.5µg/ml of dimeric rsFcγRIIIa (V158 form) or FACS-buffer and incubated for 30 min on ice followed by 1/500 streptavidin-APC (or anti-hIgG-Fc labelled with fluorescein isothiocyanate for confirmation of ACE2-Fc opsonization) for 20 min on ice. The cells were washed, resuspended in FACS buffer and analyzed on a Canto II flow cytometer (Becton Dickinson).

##### Complement fixation immunoassay for ACE2-Fc

Ninety-six well flat-bottom MaxiSorp Nunc plates (ThermoFischer Scientific) were coated with 5 μg/ml Avidin in PBS overnight, blocked, and then incubated with either two-fold dilution of biotinylated RBD (69) or 2.5 μg/ml in 0.1% casein for 1 hour at RT. The ACE2-Fc proteins were then added over the indicated concentration range. In experiments to measure C5b-C9 fixation, the plates were incubated with 10% fresh human serum for 30 minutes at RT followed by 1/2000 dilution of rabbit anti-C5b-C9 (Millipore) for 1 hour at RT, washed and then incubated with goat anti-rabbit IgG conjugated to HRP (Millipore) at 1/2000 dilution for 1 hour at RT, followed by TMB substrate for 15-20 minutes at RT (70). Reactivity was stopped using 1 M sulfuric acid and absorbance was measured at 450 nm. Test samples and reagents were prepared in PBS 0.1% (w/v) casein and plates washed thrice between each step using PBS, 0.05% (v/v) Tween 20. Samples were tested in duplicate and corrected for background reactivity using negative control wells from which ACE2-Fc proteins were omitted. The mean and SEM from independent experiments are shown.

#### Complement dependent cytotoxicity

CDC was measured by opsonizing Ramos-S cells as above (5x10^6^ cells/ml in 25µl in PBS/BSA/G for 30 min on ice) before resuspending in 1/3 diluted normal human serum for 30 min at 37°C. Cells were washed twice with PBS and the dead cells were enumerated by staining with 1/500 Zombie green (BioLegend) before fixing with 2% paraformaldehyde in PBS and analysis on a Canto II flow cytometer.

#### FcγRIIIa-NF-κB-RE nanoluciferase reporter assay

This assay used IIA1.6/FcR-γ/FcγRIIIa V158 cells expressing a NF-κB response element driven nanoluciferase (NanoLuc, pNL3.2.NF-κB-RE[NlucP/NF-κB-RE/Hygro], Promega N111) and was performed essentially as described previously (67). Briefly, Ramos cells expressing the Spike-IRES-orange2 were used as target cells and were incubated with agonists and the FcγRIIIa/NF-κB-RE reporter cells for 5h before measurement of induced nanoluciferase with Nano-Glo substrate (Promega).

#### RBD variants and coronavirus S multiplex ACE2-Fc inhibition assay

A custom coronavirus multiplex array (71) was performed using SARS-S1 subunit (S1N-S52H5, Acrobiosystems), SARS-CoV-2 S1 (40591-V08B1) and HCoV NL63 S1 and S2 subunits (40604-V08B, Sino Biological), NL63 S trimer [100788, bpsbioscience], and hexahistidine tagged RBD WT (SARS CoV-2, isolate Wuhan-Hu-1, NCBI Reference Sequence: YP_009724390.1, aa residues 319-541 (72),) and 24 variants identified from the GISAID RBD surveillance repository (71). TrACE2-Fc was biotinylated using EZ-Link^®^ Sulfo-NHS-LC-Biotin (ThermoFisher Scientific) according to the manufacturer’s instructions. Biotinylated trACE2-Fc (70 nM) was incubated with a concentration series, eight two-fold dilutions from 282 nM, of unlabelled trACE2-Fc, flACE2-Fc, EflACE2-Fc fusion proteins or the inhibitory human mAb S35 (AcroBiosystems) and binding to RBD or S proteins coupled to beads was determined using first Streptavidin, R-Phycoerythrin Conjugate (SAPE) (Thermo Fisher) at 4µg/ml (1 h), followed by 10µg/ml of R-Phycoerythrin, Biotin-XX Conjugate (Thermo Fisher) (1 h) and multiplex analysis. Apparent IC50 (nM) values are indicated from curve fits.

#### Modelling of ACE2-Fc decoy proteins

**Figure 1:**
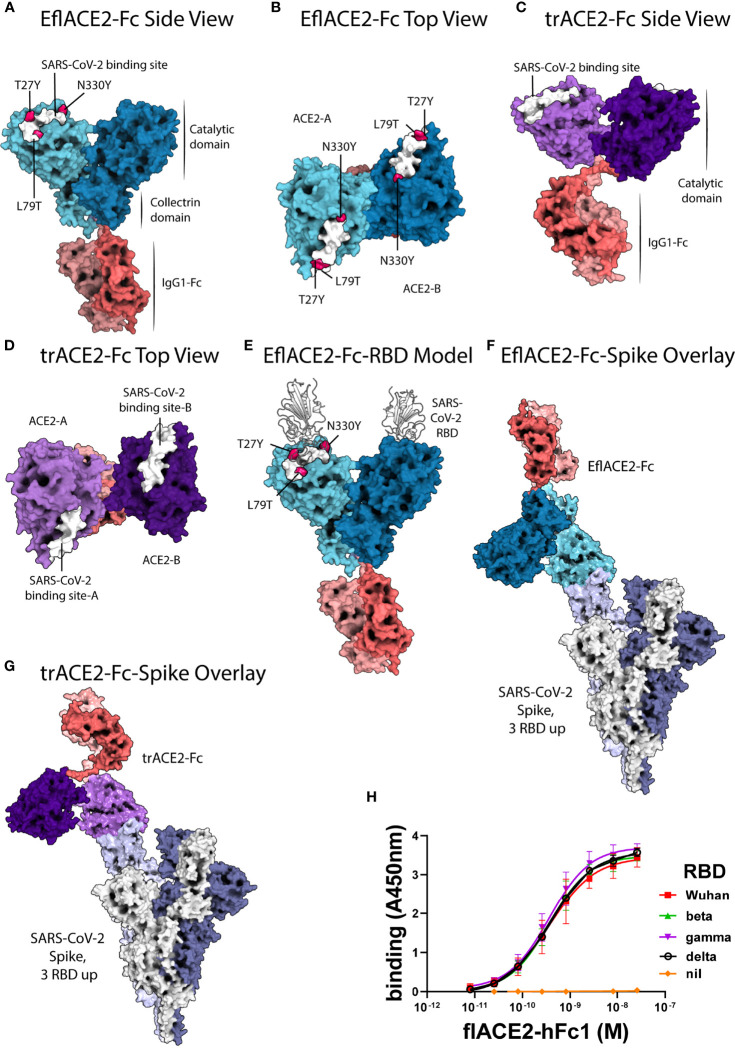
ACE2-Fc protein modelling and the interaction with SARS-CoV-2. **(A-E)** SARS-CoV-2 spike RBD binding footprint in white. The flACE2-Fc comprised a full-length ACE2 ectodomain (aa 19-740) fused to human IgG1-Fc. **(A, B)** EflACE2-Fc, having improved RBD binding is a variant of flACE2-Fc wherein three-point mutations, T27Y, L79T and N330Y, have been incorporated into ACE2 component (43) to enhance binding affinity to SARS-CoV-2 spike RBD. **(C, D)** The truncated ACE2 ectodomain (aa 19-615) was fused to human IgG1 Fc generating the trACE2-Fc fusion protein. **(E)** HADDOCK model of SARS-CoV-2 spike RBD binding to EflACE2 SARS-CoV-2 spike RBD shown in cartoon representation in white. **(F)** EflACE2-Fc shown overlayed on, and aligned by, ACE2 residues 19-614 of the 7VXM cryo-EM complex of SARS-CoV-2 spike and ACE2. **(G)** trACE2-Fc shown overlayed on, and aligned by, ACE2 residues 19-614 of the 7VXM cryo-EM complex of SARS-CoV-2 Spike and ACE2. In **(F, G)**, Positioning of the ACE2 dimer and Fc disulfides respectively indicate the ACE2-Fc constructs are unlikely to bind multiple RBD on a single spike trimer. **(H)** ACE2-Fc binding to variant SARS-CoV-2 spike RBD. The flACE2-Fc-WT fusion protein binds to the ancestral Wuhan RBD, and the beta, gamma and delta VOC RBDs with equivalent EC_50_ values. Plotted values are mean ± SD, n = 3, except for delta RBD n = 2. Agonist versus response curve fitting EC_50_ ranged from 0.31 to 0.40 nM.

Alphafold v2.2 (73, 74) was run on the EflACE2-Fc sequence using five models and specifying two homo-oligomers. The output of this recapitulated the observed structure of the ACE2 homodimer (PDB ID: 6M17, ACE2 residues 19-729) with an RMSD of 1.378 Å, however, the IgG1-Fc domains did not pair. The IgG1-Fc plus the G4S linker and collectrin domain of ACE2 (residues 615-729) was therefore run on Alphafold v2.2 specifying two homo-oligomers and an output of five models. Of these, one model showed correctly paired IgG1 Fc domains and a collectrin domain folded as in the full-length ACE2 structure (PDB ID: 6M17) with an RMSD of 0.718 Å. Superimposition of the collectrin domains of the model with the ACE2 homodimer and that with the paired IgG1-Fc allowed reconstruction for the complete EflACE2-Fc sequence. Positioning of the linkers was manually modelled based on the human B12 IgG crystal structure (PDB ID: 1HZH) to allow the correct pairing of the Fc-hinge disulphide residues at positions 749 and 752 (**Figures 1A**, **B**). The trACE2-Fc structure was modelled manually on the EflACE2-Fc model, maintaining the relative position of the ACE2 catalytic domains as in the full-length homodimer. Coordinate files are available from the authors on request.

Docking of the SARS-CoV-2 spike RBD to the EflACE2-Fc construct was modelled using HADDOCK v2.4 (75, 76) and the best model from the top scoring cluster was taken, having a HADDOCK score of -151 ± 4.2 and an RMSD from the overall lowest energy structure of 0.7 Å ± 0.5 (**Figure 1E**). The SARS-CoV-2 spike RBD and ACE2 catalytic domain (residues 19-614) had an overall RMSD of 2.733 Å from the observed SARS-CoV-2 binding to native ACE2 (PDB ID: 6M0J), with the SARS-CoV-2 and ACE2 chains aligning more closely with RMSDs of 0.400 Å and 1.400 Å respectively. A HADDOCK SARS-CoV-2 spike RBD docking model generated using an Alphafold prediction of the native ACE2 structure aligned similarly with the observed structure (PDB ID: 6M0J) with an RMSD of 2.940 Å, and overlayed the EflACE2-Fc structure with an RMSD of 0.573 Å.

*Data and Statistical analysis* used the Prism software package (GraphPad Software 9.0.2, San Diego, CA). Curve fitting to agonist(inhibitor) response curves for EC_50_ (IC_50_) determination and ANOVA with multiple comparisons tests were used as indicated in the Figure legends.

## Results

A series of ACE2-Fc fusion proteins (**Table 1**) were produced and analyzed for improved capacity to neutralize SARS-CoV-2 infection and to enhance or transform Fc-dependent effector functions attributed normally to the mechanisms of action of antibodies. Three versions of the ACE2 ectodomain were fused to the human IgG1 Fc portion. The first ACE2 fusion comprised the full length ACE2 ectodomain (flACE2-Fc, aa 19-740), including both the catalytic and collectrin domains (**Figure 1A**) and the second, an enhanced full length ACE2 ectodomain, EflACE2-Fc, with enhanced binding to SARS-CoV-2 S protein-RBD resulting from three amino acid mutations in the RBD binding site of the ACE2 protein (T27Y, L79T and N330Y) (43) and the third comprised a truncated ectodomain (trACE2-Fc, aa 19-615) containing the ACE2 catalytic domain but lacking the collectrin domain. Models of the trACE2-Fc (**Figures 1C**, **D**) and EflACE2-Fc (**Figures 1A**, **B**) decoy proteins were generated using Alphafold 2 (73, 74) and EflACE2-Fc was docked to SARS-CoV-2 spike RBD (**Figure 1E**). A comparison of the RBD docked to the Alphafold prediction of the EflACE2 and native ACE2 structures aligned similarly with the observed structure (PDB ID: 6M0J) with an RMSD of 2.733 Å and 2.940 Å respectively, and overlayed the RBD-EflACE2-Fc docked structure with an RMSD of 0.573 Å. This indicates that the affinity enhancing mutations do not impact the docking position of the SARS-CoV-2 spike RBD using this modelling approach. To evaluate the interaction with trimeric spike and assess the relative distance between ACE2 catalytic domains and adjacent RBD, the EflACE2-Fc-SARS-CoV-2 spike RBD or the trACE2-Fc-SARS-CoV-2 spike RBD model was overlayed on the RBD of chain A of the observed Spike-ACE2 complex structure (PDB ID: 7VXM) (**Figures 1F**, **G**). This showed that the ACE2 dimer in the EflACE2-Fc construct is not able to bind adjacent RBD on a single spike trimer due to distance restraints. Though the two ACE2 catalytic domains in the trACE2-Fc construct are likely not dimeric, through the lack of a collectrin domain (36), restraints imposed by disulphide bonding at the N-terminus of the Fc similarly act to restrict the distance between the ACE2 domains and likely also prevent binding to adjacent RBD for this construct (**Figure 1G**).

**Table 1:** ACE2 proteins used in this study.

Protein name	ACE2 ectodomain form(amino acid sequence)	ACE2 modification	Fc modification (IgG1 EU numbering)
trACE2	Truncated ACE2 (aa 19-615)	Not modified	N/A*
trACE2-Fc WT	Truncated ACE2 (aa 19-615)	Not modified	Not modified
trACE2-Fc-H429F	Truncated ACE2 (aa 19-615)	Not modified	His 429 Phe
trACE2-Fc-H429Y	Truncated ACE2 (aa 19-615)	Not modified	His 429 Tyr
trACE2-Fc-E430G	Truncated ACE2 (aa 19-615)	Not modified	Glu 430 Gly
trACE2-Fc-*kif*	Truncated ACE2 (aa 19-615)	Modified glycans	Modified glycan at Asn 297
flACE2-Fc-WT	Full length ACE2 (amino acids 19-740)	Not modified	Not modified
flACE2-Fc-H429F	Full length ACE2 (amino acids 19-740)	Not modified	His 429 Phe
flACE2-Fc-H429Y	Full length ACE2 (amino acids 19-740)	Not modified	His 429 Tyr
flACE2-Fc-E430G	Full length ACE2 (amino acids 19-740)	Not modified	Glu 430 Gly
EflACE2-Fc-WT	Full length ACE2 (amino acids 19-740)	Thr 27 Tyr Leu 79 Thr Asn 330 Tyr	Not modified
EflACE2-Fc-H429F	Full length ACE2 (amino acids 19-740)	Thr 27 Tyr Leu 79 Thr Asn 330 Tyr	His 429 Phe
EflACE2-Fc-H429Y	Full length ACE2 (amino acids 19-740)	Thr 27 Tyr Leu 79 Thr Asn 330 Tyr	His 429 Tyr
EflACE2-Fc-E430G	Full length ACE2 (amino acids 19-740)	Thr 27 Tyr Leu 79 Thr Asn 330 Tyr	Glu 430 Gly

*N/A, not applicable as no Fc present i.e truncated ACE2 ectodomain only.

The key rationale for the development of a ACE2 decoy antiviral protein as a biosecurity agent against a future pandemic is the presumption it will similarly inhibit SARS-CoV-2 variants and ACE2 tropic coronaviruses generally. Indeed, using an ELISA flACE2-Fc bound near equally to both the ancestral RBD and RBD from the beta, gamma and delta VOCs (**Figure 1H**).

**Figure 2:**
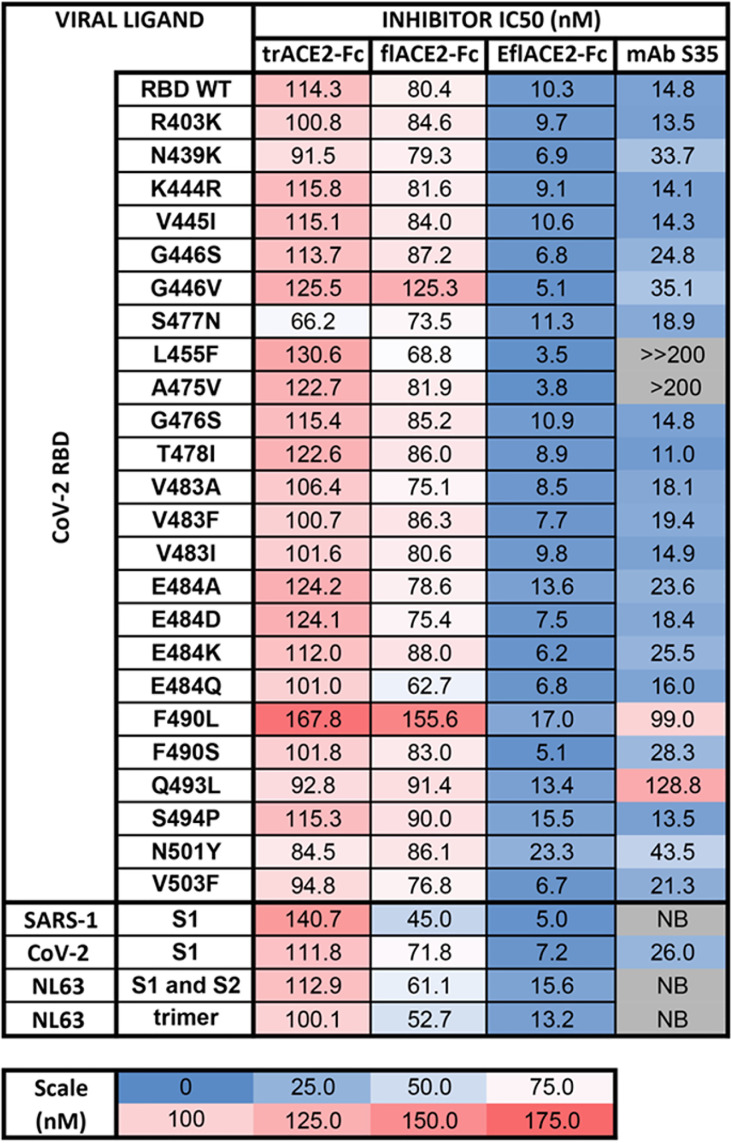
Human ACE2-Fc decoy proteins broadly inhibit binding to RBD variants and S from variants and related Sarbecoviruses. Biotinylated trACE2-Fc was incubated with a concentration series of unlabelled trACE2-Fc, flACE2-Fc, EflACE2-Fc fusion proteins or the inhibitory human mAb S35. Binding to RBD or S proteins coupled to beads was determined. Apparent IC50 (nM) values are indicated. NB, no binding.

The activity of ACE2-Fc against SARS-CoV-2 variants was further addressed using a bead array. The inhibition of binding of biotinylated trACE2-Fc to an established array of 24 SARS-CoV-2 spike RBD variants (71) by unlabeled ACE2-Fc decoys was examined. Inhibition of binding to RBD-WT followed the hierarchy trACE2-Fc-WT < flACE2-Fc-WT < EflACE2-Fc-WT (IC_50_ = 114, 80, 10 nM respectively) with effective inhibition of binding to all the individual RBD variants reached, with IC_50_ values within two-fold of that observed with the ancestral RBD (**Figure 2**). Thus, across the array of RBD variants the average IC_50_ values (110 ± 4; 86 ± 4 nM; 9.5 ± 0.9 nM) simply replicated this hierarchy of increasing neutralization potency over trACE2-Fc-WT as variants with increased affinity for ACE2 have equivalent increased susceptibility to inhibition by ACE2, including the N439K, S477N and E484K RBDs and other variants associated with escape from neutralizing antibodies (11, 54, 77). This contrasted sharply with the neutralizing mAb S35 where binding to the L455F and A475V RBD variants was abrogated. Furthermore, the decoy proteins were also effective inhibitors of binding to the spike proteins of the SARS and NL63 beta-coronaviruses (**Figure 2**). This illustrates the intrinsic resistance of ACE2 based antiviral decoys to escape by spike mutation and their applicability to other viruses that also use ACE2 for entry.

**Figure 3:**
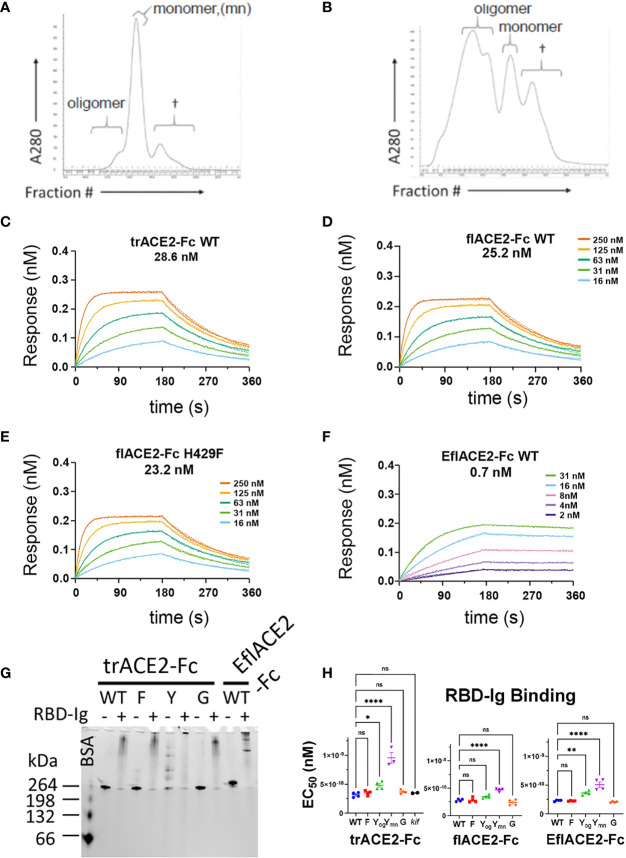
Characterization of engineered human flACE2-Fc and trACE2–Fc fusion proteins. **(A)** Size-exclusion chromatography (SEC) of IEX fractions containing flACE2-Fc-WT using a Superose 6 column, with oligomeric, monomeric forms and low mw impurities (†) indicated; and **(B)** SEC of IEX fractions containing flACE2-Fc H429Y, showing the high proportion of oligomeric species. **(C–F)** Biolayer interferometry (BLI) analysis of ACE2-Fc proteins which were immobilized on anti-human Fc (BLI) sensors and reacted with the indicated concentrations of RBD. The dissociation constants, KD (nM), are derived from global fitting of the association and dissociation curves to a Langmuir binding model. The ACE2-Fc proteins were, **(C)** trACE2-Fc WT **(D)** flACE2-Fc WT, **(E)** flACE2-Fc H429F and **(F)** the RBD binding-enhanced triple mutant of ACE2 fused to Fc; EflACE2-Fc WT (representative of n = 2 independent experiments). **(G)** Native Gel-shift analysis of ACE2-Fc proteins (1 mg, ~ 5 pmol) alone or combined with SARS-CoV-2 spike RBD-Ig (0.5 mg, ~ 5 pmol) and analyzed by native PAGE. The resulting shift in size of the proteins in the mixtures demonstrated the formation of ACE2-Fc: Cov2-RBD complexes. **(H)** Binding of different formats of ACE2-Fc-WT, and their Fc variants to immobilized RBD-Ig was determined by ELISA. EC_50_ (nM) values are from agonist versus response curve fits,mean ± SD, n is indicated by individual symbols for each independent experiment. One-way ANOVA with Dunnett’s multiple comparisons test, p > 0.05 (ns), ≤ 0.05 (*), ≤0.01 (**), ≤ 0.0001 (****).

In addition to fusion to wild-type (WT) IgG1-Fc, these ACE2 formats were also fused to a Fc carrying novel substitutions of histidine 429 (Eu numbering) with phenylalanine (H429F) or tyrosine (H429Y), or in the adjacent residue, a known IgG hexamerising mutation E430G (78, 79). A glycan-modified form of trACE2-Fc was also produced in the presence of the mannosidase inhibitor kifunensine (trACE2-Fc-*kif*). The recombinant ACE2-Fc fusion proteins were purified first by anion exchange followed by size exclusion chromatography (SEC) and comprised largely a single species by SEC (**Figure 3A**) except the H429Y mutant Fc proteins which in all formats were resolved by SEC as oligomeric and monomeric species (**Figure 3B**).

Fc modification did not affect the intrinsic affinity for the SARS-CoV-2 spike RBD (e.g. trACE2-Fc-WT, K_D_ = 28.6nM; flACE2-Fc-WT, 25.2 nM; flACE2-Fc-H429F, K_D_ = 23.2 nM, **Figures 3C-E**) which was comparable with that of the reported affinity 22nM for the flACE2 (43). As expected, the EflACE2-Fc WT protein with the enhanced RBD-binding mutant ACE2 domain showed a ~30-fold increase in affinity to K_D_ = 0.7 nM (**Figure 3F**) (43) compared to the flACE2-Fc.

Native PAGE (N-PAGE) analysis showed that ACE2-Fc WT fusion proteins migrate as a single species, at ~ 260 kDa for trACE2-Fc and at > 260 kDa for the flACE2-Fc and EflACE2-Fc fusion proteins, reflecting the additional presence of the collectrin domain (**Figure 3G**). Notably the ACE2-Fc-H429Y variants (e.g. trACE2-Fc-H429Y Fc, **Figure 3G**, 5^th^ trACE2-Fc lane “Y”) migrated in N-PAGE as several distinct higher molecular weight oligomer species, that were not apparent in denaturing SDS-PAGE, i.e. these comprise non-covalent oligomers. N-PAGE shift analysis showed that the normal and enhanced ACE2 (e.g. trACE2-Fc and EflACE2-Fc) proteins, and the Fc mutants, had high-specific binding activity for SARS-CoV-2 spike RBD, visualized by their shift to high molecular weight complexes following interaction with SARS-CoV-2 spike RBD-Ig (RBD-Ig “+” lanes, **Figure 3G**). When quantified by ELISA the ACE2-Fc proteins bound the bivalent ligand RBD-Ig with subnanomolar avidity and were unaffected by mutation of the Fc, excepting the oligomer forming H429Y Fc mutants which exhibited weaker binding (**Figure 3H**).

**Figure 4:**
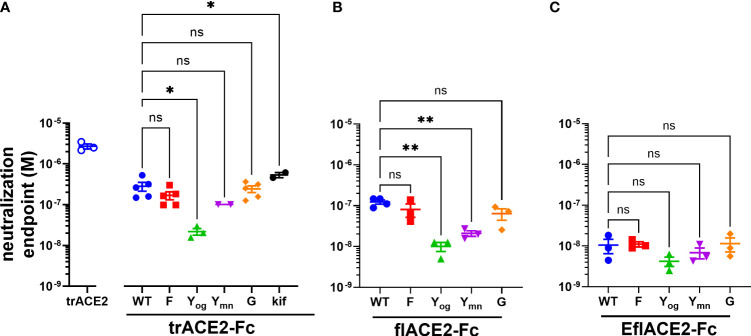
**(A–C)** SARS-CoV-2 neutralization potency of ACE2-Fc fusion proteins is increased by both the ACE2 scaffold and the H429Y Fc mutation. Neutralization potencies of the ACE2 enzymatic ectodomain polypeptide (trACE2) and the three formats of ACE2-Fc-WT fusion and variant proteins were determined by titration of the cytopathic effect to endpoint in a micro-neutralization assay. The fusion proteins were **(A)** trACE2-Fc-WT, **(B)** flACE2-Fc-WT and **(C)** EflACE2-Fc-WT, incorporating triple mutation of ACE2 engineered (43) for enhanced affinity to RBD and their Fc variants (Eu numbering), E430G, G; H429F, F; H429Y oligomers on SEC, Yog; and H429Y monomers on SEC, Ymn. A further variant trACE2-Fc fusion protein is the glycan-modified trACE2-Fc-kif produced in the presence of kifunensine. Mean ± SEM, one-way ANOVA with Dunnett’s multiple comparisons test, p < 0.05 (ns), ≤ 0.05 (*), ≤ 0.01 (**), independent experiments (n) are indicated as individual symbols.

The antiviral activities of the ACE2-Fc fusion proteins were determined in a microneutralization assay using SARS-CoV-2 infection of Vero cells (64) where the EC_50_ endpoint corresponds to neutralization of ~99% of the inoculum virions (66). The SARS-CoV-2 neutralization endpoint (EC_50_ 2.70 µM) of the unfused truncated ectodomain (trACE2 alone) was improved ~10-fold by its fusion with the unmodified wildtype Fc region of IgG1 (trACE2-Fc-WT, EC_50_ 283 nM), consistent with its improved binding avidity (**Figure 4A**). In accord with its increased intrinsic affinity for the RBD (**Figure 3F**), the EflACE2-Fc-WT (EC_50_ 11 nM) was a further ~11 and 25-fold more inhibitory than the unmodified flACE2-Fc-WT (EC_50_ 124 nM) and trACE2-Fc-WT respectively (**Figures 4A**–**C**). Thus overall, EflACE2-Fc-WT (**Figure 4C**) was ~240-fold more active in virus neutralization than trACE2 alone (**Figure 4A**).

Of the five Fc modifications, the oligomeric (og) form of the H429Y Fc mutants fused with any ACE2 format, consistently displayed superior neutralization activity within its ACE2 format class. Thus, the oligomeric trACE2-Fc-H429Y_og,_ isolated by SEC, had a neutralization activity (EC_50_ 21.9 nM) that was 13-fold improved over the monomeric trACE2-Fc-WT (EC_50_ 283nM, **Figure 4A**). Similarly, flACE2-Fc-H429Y_og_ (EC_50_ 10.0 nM) showed greater potency than flACE2-Fc-WT (EC_50_ 124 nM) (**Figure 4B**). Indeed, it was equivalent in neutralization activity to the EflACE2-Fc WT neutralization (EC_50_ 10.6 nM). Finally, the most potent inhibitor, EflACE2-Fc-H429Y_og_ (EC_50_ 4.23 nM) (**Figure 4C**), was ~ 600-fold more active than the monovalent trACE2 (**Figure 4A**). This improved neutralization by the H429Y decoy contrasted with the H429F and the E430G modifications which did not significantly alter SARS-CoV-2 neutralization activity in any ACE2-Fc format (**Figures 4A-C**). As a comparator the laboratory equivalent of the therapeutic mAb REGN 10933 (casirivimab) had an EC_50_ of 3.6 nM, (n = 2).

The Fc receptors of leukocytes and serum complement provide the two major effector systems harnessed normally by the Fc portion of antibodies. FcγR functions, which may include ADCC, phagocytosis and clearance of opsonized viruses are important antiviral effector mechanisms and are increasingly found to play a protective role during SARS-CoV-2 infection (57, 58, 60, 61, 80, 81). The interaction of FcγRIIIa with the ACE2-Fc fusion proteins was evaluated by flow cytometry using Ramos cells expressing SARS-CoV-2 spike protein (Ramos-S cells) opsonized with the different formats of ACE2-Fc. Dimeric recombinant soluble FcγRIIIa (68) bound the Fc-WT and H429F and E430G mutant fusion proteins within each ACE2 format class near equivalently. However, the H429Y mutation of the Fc largely ablated FcγR binding in all the ACE2-Fc formats (**Supplementary Figure S1A**). The loss of FcγRIIIa binding was not due to lack of opsonization of the Ramos-S cells by the H429Y variants as all ACE2-Fc proteins showed similar binding of the SARS-CoV-2 spike protein on the cell surface (**Table 2**).

**Table 2:** Flow cytometric analysis of Ramos-S cells by opsonized ACE2-Fc proteins*.

	WT	H429F	H429Y_mn_	E430G	*kif*
**trACE2-Fc**	15455 (1.00†)	16268 (1.05)	11586 (0.75)	16730 (1.08)	16382 (1.06)
**flACE2-Fc**	17246 (1.00)	16887 (0.98)	12496 (0.72)	17286 (1.00)	ND
**EflACE2-Fc**	19576 (1.00)	20472 (1.05)	15065 (0.77)	20961 (1.07)	ND

*ACE2-Fc and Fc variant fusion proteins (5 µg/ml) were reacted with Ramos-S cells and binding determined by flow cytometry.

†Median fluorescence intensity value (normalized to WT). ND, not determined.

**Figure 5:**
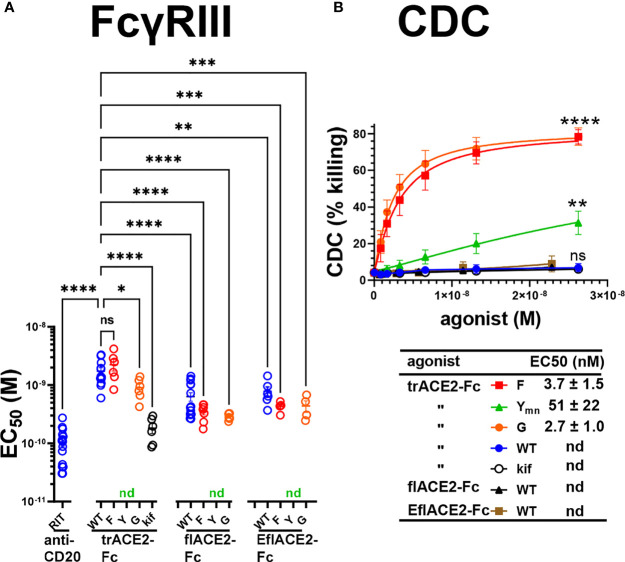
FcγR and complement dependent effector functions of the ACE2-Fc decoy proteins. **(A)** Activation of FcγRIIIa by ACE2-Fc proteins. ACE2-Fc proteins activated FcγRIIIa, except for the Fc H429Y mutants which failed to stimulate in any ACE2 format either as oligomeric or monomeric forms. Ramos-S target cells were opsonized with trACE2-Fc, flACE2-Fc and EflACE2-Fc, WT and Fc variants, including H429F, F; H429Y, Y; E430G, G or trACE2-Fc*-kif*, produced from trACE2-Fc WT in 293Expi cells in the presence of the mannosidase inhibitor kifunensine. In some experiments Ramos-S target cells were separately opsonized with Rituximab, RIT. These opsonized targets were incubated with FcγRIIIa/NF-κB-RE nanoluciferase reporter cells and FcγRIIIa activation measured by the induction of nanoluciferase (RLU). Activation data (**Supplementary Figures S1B, C**) were fitted to agonist response curves to estimate EC_50_(nM); nd, not determined as there was insufficient activity for the data to be fitted. EC_50_ values from the curve fits are shown. Mean ± SEM, n is indicated by individual symbols for each independent experiment, one-way ANOVA with Dunnett’s multiple comparisons test, comparing to trACE2-Fc WT. p > 0.05 (ns), ≤ 0.05 (*), ≤ 0.01 (**), ≤ 0.001 (***), ≤ 0.0001 (****). **(B)** H429F, and E430G Fc mutant ACE2-Fc proteins are potent mediators of complement lysis of SARS-CoV-2 S expressing cells. Flow cytometric analysis of complement-dependent cytotoxicity (CDC) of opsonized Ramos-S cells was determined in the presence of a 1/3 dilution of a pool of normal human serum (from >5 individuals) as a source of complement. Plots are mean ± SEM, n = 3 independent experiments. Two-way ANOVA with Dunnett’s multiple comparisons test comparing to trACE2-Fc-WT for main column effect, p > 0.05 (ns), ≤ 0.01 (**), ≤ 0.0001 (****). EC_50_ (nM) values are mean ± SEM each from 3 curve fits.

Next, FcγRIIIa activation by ACE2-Fc fusion proteins was evaluated as a validated surrogate of ADCC (67). FcγRIIIa was activated by Ramos-S cells opsonized with a Fc-WT fusion of any ACE2 format (**Figure 5A**). The flACE2-Fc-WT induced FcγRIIIa-mediated activation of the reporter cell at 2.7-fold lower concentration than the trACE2-Fc-WT (EC_50_ 1.7 nM) (**Figure 4A**, p < 0.0001, **Supplementary Figures S1B**, **C**), indicating that inclusion of the ACE2 collectrin domain, improved FcγRIIIa activation. Notably, the increased affinity of the EflACE2-Fc WT for SARS-CoV-2 spike protein, did not increase FcγRIIIa activation above that of flACE2-Fc WT (**Figure 5A**).

However, the most potent FcγRIIIa activation was achieved following glycan-modification by kifunensine (82) during the production of the trACE2-Fc. Thus, despite the lower activity of the trACE2-Fc format, FcγRIIIa activation by trACE2-Fc-*kif* exceeded that of the flACE2-Fc and EflACE2-Fc and approached that of the therapeutic anti-CD20 mAb rituximab used as a comparator on the CD20^+^ Ramos-S cells (**Figure 5A**; **Supplementary Figure S1B**). Thus, the hierarchy of FcγRIII activation by the proteins was trACE2-Fc-*kif* > EflACE2-Fc WT ~ flACE2-Fc- WT > trACE2-Fc-WT.

In accord with the FcγRIIIa binding data (**Supplementary Figure S1A**), modification of ACE2-Fc decoys by the H429F or E430G mutation had only modest effects on FcγRIIIa activation (**Figure 5A**; **Supplementary Figures S1B**, **C**). In contrast, the H429Y mutation in all ACE2-Fc formats ablated FcγRIIIa activation of cells which is consistent with their abrogated binding to FcγRIIIa (**Supplementary Figure S1A**). Thus, while enhancing virus neutralization, the H429Y modified Fc in trACE2-Fc, flACE2-Fc and EflACE2-Fc formats were largely inactive in FcγR binding and consequently unable to activate cells *via* FcγRIIIa (**Figure 5A**).

The second major Fc-dependent effector system is the classical complement pathway. The activation of complement by the ACE2-Fc proteins was tested initially by ELISA for the capacity to fix complement components C5b-9 and then to mediate complement-dependent killing of cells expressing SARS-CoV-2 spike protein. The fixing of C5b-9 (**Supplementary Figure S1D**) which forms the membrane attack complex, was achieved by all Fc fusions but was enhanced by both the H429F Fc mutation and the hexamerising E430G mutation of trACE2-Fc compared to unmodified Fc-WT. Despite the H429Y mutated Fc preforming oligomers, which might be anticipated to confer superior complement fixation, this was not apparent and the trACE2-Fc-H429Y oligomer form, showed similar C5b-9 fixation as the trACE2-Fc-WT and glycomodifed trACE2-Fc-*kif*, (**Supplementary Figure S1D**).

Despite the ELISA showing that the trACE2-Fc-WT fusions with an unmodified Fc fix C5b-9, analysis of cell killing showed that the unmodified Fc-WT fusion proteins of any ACE2 format failed to mediate significant CDC (**Figure 5B**). In stark contrast to this CDC inactivity, both the H429F and the hexamerising E430G Fc mutants of trACE2-Fc fusion proteins were remarkably active in mediating complement lysis of Ramos-S cells (**Figure 5B**). The monomeric form of trACE2-Fc-H429Y_mn_ was active, although substantially less potent than the H429F mutant. H429 in the Fc is thus a site for modification that remarkably potentiates the Fc’s capacity for stimulating complement-mediated target lysis.

## Discussion

In this study we examined different antiviral functions of ACE2-Fc virus decoy proteins. As in IgG antibodies, the Fc-region drives the effector responses mediated by the IgG-Fc fusion proteins. *In vivo* SARS-CoV-2 challenge models (57–61) have found Fc immune functions of antibodies decreased virus load, spread from nasal tissue to major organ systems, cytokine storm and inflammation, and mortality. In contrast, ablating Fc function resulted in increased disease severity or mortality (58–60, 83). Recently a non-neutralizing human mAb with Fc-enhanced ADCC activity conferred partial protection in a SARS-CoV-2 infection model and contributed to complete protection in combination with a neutralizing mAb (80). Indeed, ADCC potency is an indicator of humoral responses that protect against severe disease in humans (81). While complement activation features in the pathophysiology of severe COVID-19 it is likely to be initially protective (84) and is an identified function of anti-SARS-CoV-2 therapeutic antibodies (85). The Fc portion is thus an important element to optimize for the development of ACE2-Fc as an anti-SARS-CoV-2 antiviral molecule and for viral entry receptors fused to Fc more generally.

Hence, we have manipulated three major antiviral activities of ACE2-Fc by modifying its Fc portion to enhance the existing decoy (neutralization) action of the ACE2 component, complement mediated killing and activation of FcγR. Firstly, the H429Y mutation, in the Fc CH3 domain outside the Fcγ receptor or complement contact sites of the CH2 domain, resulted in the formation of oligomers of the decoy protein which resulted in improved neutralization potency. The improved neutralization activity and oligomeric nature of the H429Y Fc mutant decoys mimic the polymeric antibody classes, IgA (86) and IgM (87) where avidity contributes to the efficacy of SARS-CoV-2 neutralization. Fc : Fc interactions are a recognized property of IgG antibodies (88) and their stabilization by mutation can lead to the formation of in solution oligomers (89). In contrast, the E430G modification of IgG is known to promote “on-target” oligomerization (hexamerization) of IgG (79), but did not significantly alter SARS-CoV-2 neutralization activity in any ACE2-Fc format. In contrast, the H429Y Fc mutation enhanced neutralization potency of all formats of the ACE2-Fc decoy proteins. H429Y Fc mutation in combination with the inclusion of the collectrin domain and the triple ACE2 mutations enhancing affinity for S (43), (i.e. EflACE2-Fc-H429Y) resulted in an overall 600-fold increased SARS-CoV-2 neutralization potency over that of the monomeric truncated ACE2 domain. The neutralization potency of EflACE2-Fc-H429Y (4.2 nM) was comparable to that of the laboratory equivalent of the therapeutic mAb REGN 10933 (casirivimab, 3.6 nM). A feature of the H429Y mutation was the loss of binding by FcγRIIIa. Mutations at the CH2/CH3 interface can affect low affinity FcγR binding to the Fc (90), suggesting these sites, though distant, can affect each other (91).

Secondly, the phenylalanine substitution of histidine 429 (H429F) of the ACE2-Fc proteins did not enhance neutralization but did transform CDC against S expressing targets. This improved CDC activity was like that of E430G mutated ACE2-Fc, a known “on-target” Fc-hexamerising mutation, a format optimal for C1 binding and activation (78, 79). Lastly, FcγR potency of trACE2-Fc was improved by modifying the Fc glycan (82) to enhance FcγRIII binding (92). It is likely that similar treatment of flACE2-Fc WT and EflACE2-Fc WT, or alternatively, amino acid substitution to increase affinity for FcγRIIIa (93), would similarly further improve their FcγRIII activating potency. Notably, FcγRIII activation was a little reduced for the decoy lacking the collection domain, indicating the formatting of Fc-fusion proteins can impact Fc-mediated activity.

We have demonstrated ACE2-Fc to be a potent agent against SARS-CoV-2, not only for neutralization but also for the harnessing of Fc-mediated effector functions. The exemplar Fc modifications demonstrated herein illustrate the potential for the tuning of Fc function to optimize virus neutralization, FcγR interaction and complement activation. This selection of desired functional profiles could aid the deployment of broadly effective ACE2-Fc, mAbs and other Fc therapeutics. There has been a rapid progression of multiple different SARS-CoV-2 mAbs to clinical use that is likely to herald increased deployment of mAbs clinically for infectious diseases. The optimization of Fc functions will make a significant difference to their clinical success. Furthermore, the world remains susceptible to new pandemics and vaccine escape variants. Thus, an antiviral decoy comprising optimized Fc fusion to a viral entry receptor such as ACE2-Fc, is an important option for deploying a rapid first line of defense to contain new zoonotic viral threats while vaccines, mAbs and antiviral drugs are being developed.

## Data availability statement

The raw data supporting the conclusions of this article will be made available by the authors, without undue reservation. PDB coordinate files are available from the authors on request.

## Author contributions

BW and PMH conceived and planned the experiments. BW, LK, HT, SE, EL, KC, L-JC, FM, WL, NG, SP, GH, PP, and JC performed the experiments and analyzed the data. JB, DG, LB, MvZ, AW, AC, W-HT, KS, SK, and PMH provided supervision and analyzed the data. BW and PMH wrote the manuscript with input from all other authors. All authors contributed to the article and approved the submitted version.

## Funding

The Medical Research Future Fund (MRFF 2002073) and Victorian State Government COVID research funding supported the research of AW, DG, W-HT, SK, PH, and SP, LB (1175865) with contribution from the Victorian Operational Infrastructure Support Program and Australian Government NHMRC Independent Research Institutes Infrastructure Support Scheme. NHMRC project grants supported PH, BW. (1145303). Investigator Grants are held by JB (1173046), KS (1177174) and DG (2008913) and program grants by SK (1149990), LB (1055214). DG (1117766), W-HT, SK, MZ (1117687) and AW receive National Health and Medical Research Council (NHMRC) fellowships. DG is supported by the Australian Research Council (ARC; CE140100011). NG was supported by an ARC DECRA Fellowship (DE210100705). SP and LB funded by a Heart Foundation Vanguard Grant (105798). The Melbourne WHO Collaborating Centre for Reference and Research on Influenza is supported by the Australian Government Department of Health. W-HT is a Howard Hughes Medical Institute–Wellcome Trust International Research Scholar (208693/Z/17/Z).

## Conflict of interest

Authors PMH and BW are inventors on a provisional patent filing by the Burnet Institute.

The remaining authors declare that the research was conducted in the absence of any commercial or financial relationships that could be construed as a potential conflict of interest.

## Publisher’s note

All claims expressed in this article are solely those of the authors and do not necessarily represent those of their affiliated organizations, or those of the publisher, the editors and the reviewers. Any product that may be evaluated in this article, or claim that may be made by its manufacturer, is not guaranteed or endorsed by the publisher.

## Supplementary material

Supplementary Figure 1 Human ACE2-Fc proteins activate FcγRIIIa and Complement. **(A)** FcγRIIIa binding. The ACE2-Fc WT fusion proteins and their variants (5 µg/ml) were reacted with Ramos-S cells (Ramos cells expressing spike protein) and Fc receptor binding evaluated by flow cytometry using biotinylated dimeric rsFcγRIIIa, followed by streptavidin-APC. (mean of 3 replicates). **(B, C)** Activation of FcγRIIIa. ACE2-Fc proteins are potent activators of FcγRIIIa apart from the Fc H429Y mutants which fail to stimulate FcγRIIIa in any ACE2 format. Ramos-S target cells were opsonized with **(B)** trACE2-Fc and **(C)** flACE2-Fc, WT and separately with Fc variants, including H429F, F; H429Y unfractionated, Y; H429Y oligomers, Y_og_; H429Y monomer, Y_mn_; E430G, G or trACE2-Fc *kif* produced from trACE2-Fc WT in 293Expi cells in the presence of the mannosidase inhibitor kifunensine. Ramos-S target cells were separately opsonized with Rituximab, RIT. These opsonized targets were incubated with FcγRIIIa-NF-κB-RE nanoluciferase reporter cells and FcγRIIIa activation measured by the induction of nanoluciferase (RLU). Representative activation data showing fitting to agonist response curves to determine each EC_50_ (nM) data point shown in **Figure 5A**. **(D)** ACE2-Fc fusion proteins comprising Fc regions with either of the H429F and E430G mutations, strongly fix complement C5b-9. In ELISA analysis the indicated concentration series of trACE2-Fc or its Fc variants, was bound to SARS-CoV-2 spike RBD-biotin (2.5 μg/ml) captured by plate bound avidin (2 μg/ml). Following incubation with human serum the formation of C5b-9 was determined, (mean ± SD); two independent experiments.
