# Corrigendum: Fc engineered ACE2-Fc is a potent multifunctional agent targeting SARS-CoV2

**DOI:** 10.3389/fimmu.2022.1122516

**Published:** 2023-01-10

**Authors:** Bruce D. Wines, Liriye Kurtovic, Halina M. Trist, Sandra Esparon, Ester Lopez, Klasina Chappin, Li-Jin Chan, Francesca L. Mordant, Wen Shi Lee, Nicholas A. Gherardin, Sheila K. Patel, Gemma E. Hartley, Phillip Pymm, James P. Cooney, James G. Beeson, Dale I. Godfrey, Louise M. Burrell, Menno C. van Zelm, Adam K. Wheatley, Amy W. Chung, Wai-Hong Tham, Kanta Subbarao, Stephen J. Kent, P. Mark Hogarth

**Affiliations:** ^1^Immune therapies Laboratory, Burnet Institute, Melbourne, VIC, Australia; ^2^Life Sciences, Burnet Institute, Melbourne, VIC, Australia; ^3^Department of Immunology and Pathology, Central Clinical School, Monash University, Melbourne, VIC, Australia; ^4^Department of Clinical Pathology, The University of Melbourne, Parkville, VIC, Australia; ^5^Department of Microbiology and Immunology, The Peter Doherty Institute for Infection and Immunity, The University of Melbourne, Melbourne, VIC, Australia; ^6^Infectious Diseases and Immune Defence Division, The Walter and Eliza Hall Institute of Medical Research, Parkville, VIC, Australia; ^7^Department of Medical Biology, The University of Melbourne, Melbourne, VIC, Australia; ^8^Department of Medicine, Austin Health, The University of Melbourne, Melbourne, VIC, Australia; ^9^Department of Medicine, Royal Melbourne Hospital, The University of Melbourne, Parkville, VIC, Australia; ^10^Department of Microbiology, Monash University, Clayton VIC, Australia; ^11^Department of Allergy, Immunology and Respiratory Medicine, Central Clinical School, Alfred Hospital, Melbourne, VIC, Australia; ^12^Australian Research Council Centre for Excellence in Convergent Bio-Nano Science and Technology, The University of Melbourne, Melbourne, VIC, Australia; ^13^World Health Organization (WHO) Collaborating Centre for Reference and Research on Influenza, The Peter Doherty Institute for Infection and Immunity, The University of Melbourne, Melbourne, VIC, Australia; ^14^Melbourne Sexual Health Centre and Department of Infectious Diseases, Alfred Hospital and Central Clinical School, Monash University, Melbourne, VIC, Australia

**Keywords:** coronavirus, SARS-CoV-2, COVID-19, ACE2-Fc, neutralization, antibody effector function, ADCC, complement

In the published article, there was an error in the legend for Figure 3 as published. The statistics style output rather than the significance levels was reported. The corrected legend appears below.

“Characterization of engineered human flACE2-Fc and trACE2–Fc fusion proteins. (A) Size-exclusion chromatography (SEC) of IEX fractions containing flACE2-Fc-WT using a Superose 6 column, with oligomeric, monomeric forms and low mw impurities (†) indicated; and (B) SEC of IEX fractions containing flACE2-Fc H429Y, showing the high proportion of oligomeric species. (C–F) Biolayer interferometry (BLI) analysis of ACE2-Fc proteins which were immobilized on anti-human Fc (BLI) sensors and reacted with the indicated concentrations of RBD. The dissociation constants, K_D_ (nM), are derived from global fitting of the association and dissociation curves to a Langmuir binding model. The ACE2-Fc proteins were, (C) trACE2-Fc WT (D) flACE2-Fc WT, (E) flACE2-Fc H429F and (F) the RBD binding-enhanced triple mutant of ACE2 fused to Fc; EflACE2-Fc WT (representative of n = 2 independent experiments). (G) Native Gel-shift analysis of ACE2-Fc proteins (1 μg, ~ 5 pmol) alone or combined with SARS-CoV-2 spike RBD-Ig (0.5 μg, ~ 5 pmol) and analyzed by native PAGE. The resulting shift in size of the proteins in the mixtures demonstrated the formation of ACE2-Fc: Cov2-RBD complexes. (H) Binding of different formats of ACE2-Fc-WT, and their Fc variants to immobilized RBD-Ig was determined by ELISA. EC_50_ (nM) values are from agonist versus response curve fits, mean ± SD, n is indicated by individual symbols for each independent experiment. One-way ANOVA with Dunnett’s multiple comparisons test, p > 0.05 (ns), ≤ 0.05 (*), ≤ 0.01 (**), ≤ 0.0001 (****).”

In the published article, there was an error in the legend for Figure 4 as published. The statistics style output rather than the significance levels was reported. The corrected legend appears below.

“(A-C) SARS-CoV-2 neutralization potency of ACE2-Fc fusion proteins is increased by both the ACE2 scaffold and the H429Y Fc mutation. Neutralization potencies of the ACE2 enzymatic ectodomain polypeptide (trACE2) and the three formats of ACE2-Fc-WT fusion and variant proteins were determined by titration of the cytopathic effect to endpoint in a micro-neutralization assay. The fusion proteins were (A) trACE2-Fc-WT, (B) flACE2-Fc-WT and (C) EflACE2-Fc-WT, incorporating triple mutation of ACE2 engineered (43) for enhanced affinity to RBD and their Fc variants (Eu numbering), E430G, G; H429F, F; H429Y oligomers on SEC, Yog; and H429Y monomers on SEC, Ymn. A further variant trACE2-Fc fusion protein is the glycan-modified trACE2-Fc-kif produced in the presence of kifunensine. Mean ± SEM, one-way ANOVA with Dunnett’s multiple comparisons test, p > 0.05 (ns), ≤ 0.05 (*), ≤ 0.01 (**), independent experiments (n) are indicated as individual symbols.”

In the published article, there was an error in the legend for Figure 5 as published. The statistics style output rather than the significance levels was reported. The corrected legend appears below.

“FcγR and complement dependent effector functions of the ACE2-Fc decoy proteins. (A) Activation of FcγRIIIa by ACE2-Fc proteins. ACE2-Fc proteins activated FcγRIIIa, except for the Fc H429Y mutants which failed to stimulate in any ACE2 format either as oligomeric or monomeric forms. Ramos-S target cells were opsonized with trACE2-Fc, flACE2-Fc and EflACE2-Fc, WT and Fc variants, including H429F, F; H429Y, Y; E430G, G or trACE2-Fc-*kif*, produced from trACE2-Fc WT in 293Expi cells in the presence of the mannosidase inhibitor kifunensine. In some experiments Ramos-S target cells were separately opsonized with Rituximab, RIT. These opsonized targets were incubated with FcγRIIIa/NF-κB-RE nanoluciferase reporter cells and FcγRIIIa activation measured by the induction of nanoluciferase (RLU). Activation data (Supplementary Figures S1B, C) were fitted to agonist response curves to estimate EC_50_(nM); nd, not determined as there was insufficient activity for the data to be fitted. EC_50_ values from the curve fits are shown. Mean ± SEM, n is indicated by individual symbols for each independent experiment, one-way ANOVA with Dunnett’s multiple comparisons test, comparing to trACE2-Fc WT. p > 0.05 (ns), ≤ 0.05 (*), ≤ 0.01 (**), ≤ 0.001 (***), ≤ 0.0001 (****). (B) H429F, and E430G Fc mutant ACE2-Fc proteins are potent mediators of complement lysis of SARS-CoV-2 S expressing cells. Flow cytometric analysis of complement-dependent cytotoxicity (CDC) of opsonized Ramos-S cells was determined in the presence of a 1/3 dilution of a pool of normal human serum (from >5 individuals) as a source of complement. Plots are mean ± SEM, n = 3 independent experiments. Two-way ANOVA with Dunnett’s multiple comparisons test comparing to trACE2-Fc-WT for main column effect, p > 0.05 (ns), ≤ 0.01 (**), ≤ 0.0001 (****). EC_50_ (nM) values are mean ± SEM each from 3 curve fits.”

The authors apologize for these errors and state that they do not change the scientific conclusions of the article in any way. The original article has been updated.

