# Supplementary material for: Fc engineered ACE2-Fc is a potent multifunctional agent targeting SARS-CoV2
Source: Front Immunol. 2022 Jul 28;13:889372. doi: 10.3389/fimmu.2022.889372 (PMC9369017; doi:10.3389/fimmu.2022.889372)
Supplement: Supplementary file 1 [file DataSheet_1.docx]

**Supplementary text.**

| mature "flACE2-Fc-WT" fusion polypeptide  20 30 40 50 60 70 80  *ETG*ST IEEQAKTFLD KFNHEAEDLF YQSSLASWNY NTNITEENVQ NMNNAGDKWS AFLKEQSTLA  90 100 110 120 130 140 150 160  QMYPLQEIQN LTVKLQLQAL QQNGSSVLSE DKSKRLNTIL NTMSTIYSTG KVCNPDNPQE CLLLEPGLNE IMANSLDYNE  170 180 190 200 210 220 230 240  RLWAWESWRS EVGKQLRPLY EEYVVLKNEM ARANHYEDYG DYWRGDYEVN GVDGYDYSRG QLIEDVEHTF EEIKPLYEHL  250 260 270 280 290 300 310 320  HAYVRAKLMN AYPSYISPIG CLPAHLLGDM WGRFWTNLYS LTVPFGQKPN IDVTDAMVDQ AWDAQRIFKE AEKFFVSVGL  330 340 350 360 370 380 390 400  PNMTQGFWEN SMLTDPGNVQ KAVCHPTAWD LGKGDFRILM CTKVTMDDFL TAHHEMGHIQ YDMAYAAQPF LLRNGANEGF  410 420 430 440 450 460 470 480  HEAVGEIMSL SAATPKHLKS IGLLSPDFQE DNETEINFLL KQALTIVGTL PFTYMLEKWR WMVFKGEIPK DQWMKKWWEM  490 500 510 520 530 540 550 560  KREIVGVVEP VPHDETYCDP ASLFHVSNDY SFIRYYTRTL YQFQFQEALC QAAKHEGPLH KCDISNSTEA GQKLFNMLRL  570 580 590 600 610 620 630 640  GKSEPWTLAL ENVVGAKNMN VRPLLNYFEP LFTWLKDQNK NSFVGWSTDW SPYADQSIKV RISLKSALGD RAYEWNDNEM  650 660 670 680 690 700 710 720  YLFRSSVAYA MRQYFLKVKN QMILFGEEDV RVANLKPRIS FNFFVTAPKN VSDIIPRTEV EKAIRMSRSR INDAFRLNDN  730 740 750 760 770 780 790 800  *Fc (EU numbering →) 227 237 247 257 267 277*  SLEFLGIQPT LGPPNQPPVS *GGGGS*THTCP PCPAPELLGG PSVFLFPPKP KDTLMISRTP EVTCVVVDVS HEDPEVKFNW  810 820 830 840 850 860 870 880  *287 297 307 317 327 337 347 357*  YVDGVEVHNA KTKPREEQYN STYRVVSVLT VLHQDWLNGK EYKCKVSNKA LPAPIEKTIS KAKGQPREPQ VYTLPPSREE  890 900 910 920 930 940 950 960  *367 377 387 397 407 417 427 437*  MTKNQVSLTC LVKGFYPSDI AVEWESNGQP ENNYKTTPPV LDSDGSFFLY SKLTVDKSRW QQGNVFSCSV M**H**EALHNHYT  970  *447*  QKSLSLSPGK |
| --- |
| The predicted sequence of the mature "flACE2-Fc" fusion polypeptide. The full-length (fl)ACE2 ectodomain comprises the catalytic domain and the collectrin (or neck) domain and sequence proximal to the transmembrane region. The N-terminal sequence *ETG* is predicted to remain following cleavage of the following underlined leader sequence (MGILPSPGMPALLSLVSLLSVLL MGCVAETG) upon secretion. The numbering above the fusion protein sequence utilises the numbering of ACE2, amino acid residues S19 to S740, then a linking sequence (*GGGGS*), followed by a human IgG1 Fc comprising T223-K447 (according to the EU numbering convention, labeled immediately above the C-terminal Fc sequence) and includes the CH2 and CH3 domains. The IgG1 Fc amino acid, H429, is shown in bold and underlined (EU numbering). The ACE2 ectodomain sequence matches that of Accession no. BAB40370. Also, a variant immunotherapeutic protein "EflACE2-Fc" was constructed whereby, the amino acids at positions 27, 79 and 330 were mutated to enhance binding affinity to CoV-2 S as follows: T27Y, L79T and N330Y (Chan *et al*., 2020). The IgG1 Fc sequence matches the immunoglobulin gamma 1 constant region, partial [Homo sapiens] sequence of GenBank Accession no. AXN93652.1. |

| mature "EflACE2-Fc-WT" fusion polypeptide  20 30 40 50 60 70 80  *ETG*ST IEEQAKYFLD KFNHEAEDLF YQSSLASWNY NTNITEENVQ NMNNAGDKWS AFLKEQSTTA  90 100 110 120 130 140 150 160  QMYPLQEIQN LTVKLQLQAL QQNGSSVLSE DKSKRLNTIL NTMSTIYSTG KVCNPDNPQE CLLLEPGLNE IMANSLDYNE  170 180 190 200 210 220 230 240  RLWAWESWRS EVGKQLRPLY EEYVVLKNEM ARANHYEDYG DYWRGDYEVN GVDGYDYSRG QLIEDVEHTF EEIKPLYEHL  250 260 270 280 290 300 310 320  HAYVRAKLMN AYPSYISPIG CLPAHLLGDM WGRFWTNLYS LTVPFGQKPN IDVTDAMVDQ AWDAQRIFKE AEKFFVSVGL  330 340 350 360 370 380 390 400  PNMTQGFWEY SMLTDPGNVQ KAVCHPTAWD LGKGDFRILM CTKVTMDDFL TAHHEMGHIQ YDMAYAAQPF LLRNGANEGF  410 420 430 440 450 460 470 480  HEAVGEIMSL SAATPKHLKS IGLLSPDFQE DNETEINFLL KQALTIVGTL PFTYMLEKWR WMVFKGEIPK DQWMKKWWEM  490 500 510 520 530 540 550 560  KREIVGVVEP VPHDETYCDP ASLFHVSNDY SFIRYYTRTL YQFQFQEALC QAAKHEGPLH KCDISNSTEA GQKLFNMLRL  570 580 590 600 610 620 630 640  GKSEPWTLAL ENVVGAKNMN VRPLLNYFEP LFTWLKDQNK NSFVGWSTDW SPYADQSIKV RISLKSALGD RAYEWNDNEM  650 660 670 680 690 700 710 720  YLFRSSVAYA MRQYFLKVKN QMILFGEEDV RVANLKPRIS FNFFVTAPKN VSDIIPRTEV EKAIRMSRSR INDAFRLNDN  730 740 750 760 770 780 790 800  *Fc (EU numbering →) 227 237 247 257 267 277*  SLEFLGIQPT LGPPNQPPVS *GGGGS*THTCP PCPAPELLGG PSVFLFPPKP KDTLMISRTP EVTCVVVDVS HEDPEVKFNW  810 820 830 840 850 860 870 880  *287 297 307 317 327 337 347 357*  YVDGVEVHNA KTKPREEQYN STYRVVSVLT VLHQDWLNGK EYKCKVSNKA LPAPIEKTIS KAKGQPREPQ VYTLPPSREE  890 900 910 920 930 940 950 960  *367 377 387 397 407 417 427 437*  MTKNQVSLTC LVKGFYPSDI AVEWESNGQP ENNYKTTPPV LDSDGSFFLY SKLTVDKSRW QQGNVFSCSV M**H**EALHNHYT  970  *447*  QKSLSLSPGK |
| --- |
| The predicted sequence of the mature "EflACE2-Fc" fusion polypeptide. The full-length (fl)ACE2 ectodomain comprises the catalytic domain and the collectrin (or neck) domain and sequence proximal to the transmembrane region. The N-terminal sequence *ETG* is predicted to remain following cleavage of the following underlined leader sequence (MGILPSPGMPALLSLVSLLSVLL MGCVAETG) upon secretion. The numbering above the fusion protein sequence utilises the numbering of ACE2, amino acid residues S19 to S740, then a linking sequence (*GGGGS*), followed by a human IgG1 Fc comprising T223-K447 (according to the EU numbering convention, labeled immediately above the C-terminal Fc sequence) and includes the CH2 and CH3 domains. The IgG1 Fc amino acid, H429, is shown in bold and underlined (EU numbering). The ACE2 ectodomain sequence matches that of Accession no. BAB40370. In this variant immunotherapeutic protein "EflACE2-Fc", the amino acids at positions 27, 79 and 330 were mutated to enhance binding affinity to CoV-2 S as follows: T27Y, L79T and N330Y (Chan *et al*., 2020). The IgG1 Fc sequence matches the immunoglobulin gamma 1 constant region, partial [Homo sapiens] sequence of GenBank Accession no. AXN93652.1. |

| mature "trACE2-Fc-WT" fusion polypeptide  20 30 40 50 60 70 80  *ETG*ST IEEQAKTFLD KFNHEAEDLF YQSSLASWNY NTNITEENVQ NMNNAGDKWS AFLKEQSTLA  90 100 110 120 130 140 150 160  QMYPLQEIQN LTVKLQLQAL QQNGSSVLSE DKSKRLNTIL NTMSTIYSTG KVCNPDNPQE CLLLEPGLNE IMANSLDYNE  170 180 190 200 210 220 230 240  RLWAWESWRS EVGKQLRPLY EEYVVLKNEM ARANHYEDYG DYWRGDYEVN GVDGYDYSRG QLIEDVEHTF EEIKPLYEHL  250 260 270 280 290 300 310 320  HAYVRAKLMN AYPSYISPIG CLPAHLLGDM WGRFWTNLYS LTVPFGQKPN IDVTDAMVDQ AWDAQRIFKE AEKFFVSVGL  330 340 350 360 370 380 390 400  PNMTQGFWEN SMLTDPGNVQ KAVCHPTAWD LGKGDFRILM CTKVTMDDFL TAHHEMGHIQ YDMAYAAQPF LLRNGANEGF  410 420 430 440 450 460 470 480  HEAVGEIMSL SAATPKHLKS IGLLSPDFQE DNETEINFLL KQALTIVGTL PFTYMLEKWR WMVFKGEIPK DQWMKKWWEM  490 500 510 520 530 540 550 560  KREIVGVVEP VPHDETYCDP ASLFHVSNDY SFIRYYTRTL YQFQFQEALC QAAKHEGPLH KCDISNSTEA GQKLFNMLRL  570 580 590 600 610 620 630 640  *Fc (EU numbering →)* *230* *240*  GKSEPWTLAL ENVVGAKNMN VRPLLNYFEP LFTWLKDQNK NSFVGWSTDW SPYAD*GSGSG SG*THTCPPCP APELLGGPSV  650 660 670 680 690 700 710 720  *250 260 270 280 290 300 310 320*  FLFPPKPKDT LMISRTPEVT CVVVDVSHED PEVKFNWYVD GVEVHNAKTK PREEQYNSTY RVVSVLTVLH QDWLNGKEYK  730 740 750 760 770 780 790 800  *330 340 350 360 370 380 390 400*  CKVSNKALPA PIEKTISKAK GQPREPQVYT LPPSREEMTK NQVSLTCLVK GFYPSDIAVE WESNGQPENN YKTTPPVLDS  810 820 830 840 847  *410 420 430 440 447*  DGSFFLYSKL TVDKSRWQQG NVFSCSVM**H**E ALHNHYTQKS LSLSPGK |
| --- |
| The predicted sequence of the mature "trACE2-Fc" fusion polypeptide. The N-terminal sequence ETG is predicted to remain following cleavage of the following underlined leader sequence (MGILPSPGMPALLSLVSLLSVLLMGCVAETG) upon secretion. The numbering above the fusion protein sequence utilises the numbering of ACE2, amino acid residues S19 to D615, then a linking sequence (*GSGSGSG*), followed by a human IgG1 Fc, and which, according to the EU numbering convention (labeled immediately above the C-terminal Fc sequence), comprises T223-K447. The IgG1 Fc amino acid, H429, is shown bold and underlined. The truncated ACE2 ectodomain sequence matches that of Accession no. BAB40370. The IgG1 Fc sequence matches the immunoglobulin gamma 1 constant region, partial [Homo sapiens] sequence of Accession no. AXN93652.1. |
