# Supplementary figures and images for: Fc engineered ACE2-Fc is a potent multifunctional agent targeting SARS-CoV2
Source: Front Immunol. 2022 Jul 28;13:889372. doi: 10.3389/fimmu.2022.889372 (PMC9369017; doi:10.3389/fimmu.2022.889372)

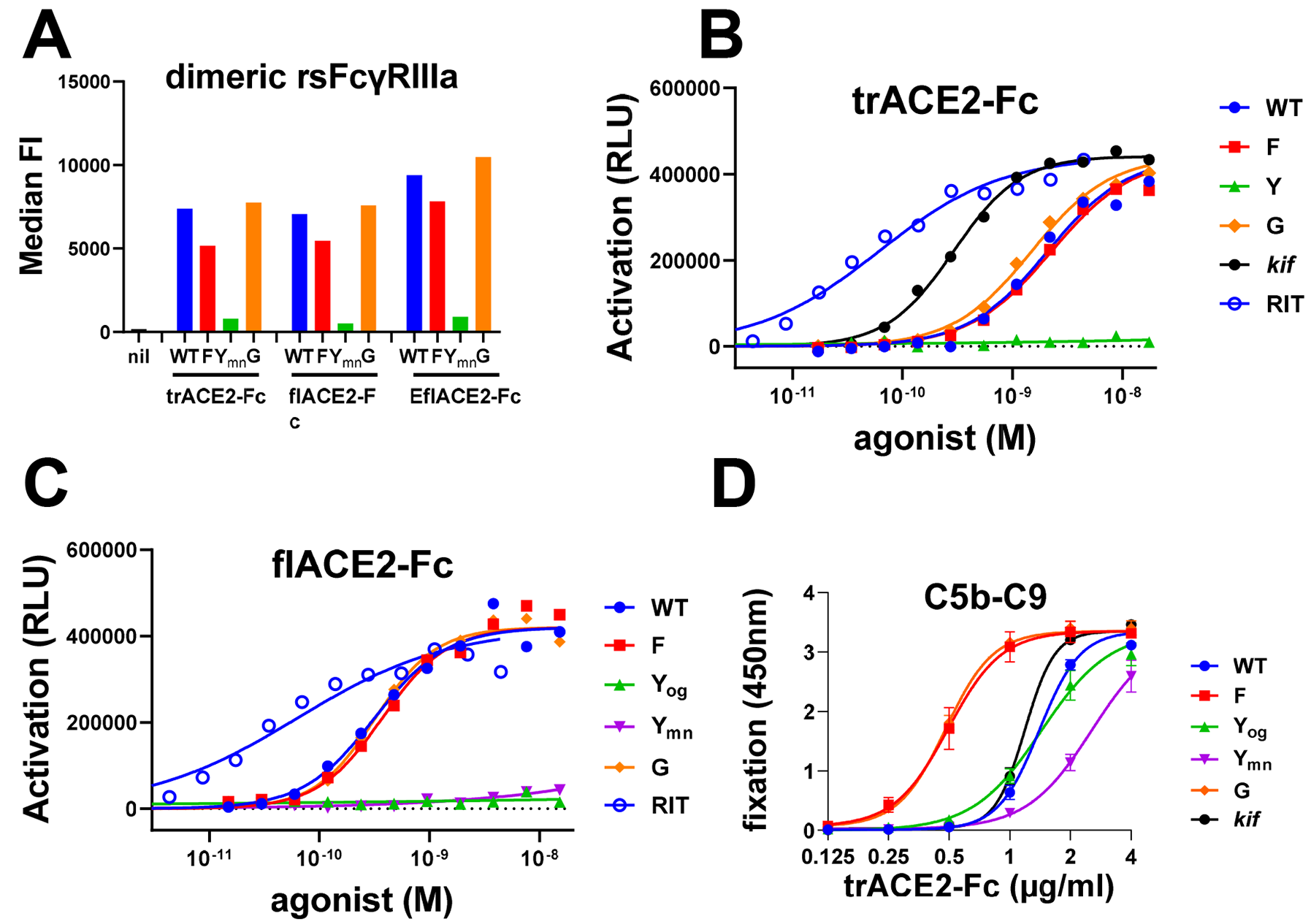

Supplement: Supplementary Figure 1 — Human ACE2-Fc proteins activate FcγRIIIa and Complement. (A) FcγRIIIa binding. The ACE2-Fc WT fusion proteins and their variants (5 µg/ml) were reacted with Ramos-S cells (Ramos cells expressing spike protein) and Fc receptor binding evaluated by flow cytometry using biotinylated dimeric rsFcγRIIIa, followed by streptavidin-APC. (mean of 3 replicates). (B, C) Activation of FcγRIIIa. ACE2-Fc proteins are potent activators of FcγRIIIa apart from the Fc H429Y mutants which fail to stimulate FcγRIIIa in any ACE2 format. Ramos-S target cells were opsonized with (B) trACE2-Fc and (C) flACE2-Fc, WT and separately with Fc variants, including H429F, F; H429Y unfractionated, Y; H429Y oligomers, Yog; H429Y monomer, Ymn; E430G, G or trACE2-Fc kif produced from trACE2-Fc WT in 293Expi cells in the presence of the mannosidase inhibitor kifunensine. Ramos-S target cells were separately opsonized with Rituximab, RIT. These opsonized targets were incubated with FcγRIIIa-NF-κB-RE nanoluciferase reporter cells and FcγRIIIa activation measured by the induction of nanoluciferase (RLU). Representative activation data showing fitting to agonist response curves to determine each EC50 (nM) data point shown in Figure 5A. (D) ACE2-Fc fusion proteins comprising Fc regions with either of the H429F and E430G mutations, strongly fix complement C5b-9. In ELISA analysis the indicated concentration series of trACE2-Fc or its Fc variants, was bound to SARS-CoV-2 spike RBD-biotin (2.5 μg/ml) captured by plate bound avidin (2 μg/ml). Following incubation with human serum the formation of C5b-9 was determined, (mean ± SD); two independent experiments. [file Image_1.tif]
